# Dual-layer spectral-detector CT for detecting liver steatosis by using proton density fat fraction as reference

**DOI:** 10.1186/s13244-024-01716-6

**Published:** 2024-08-15

**Authors:** Min Wang, Hongyu Chen, Yue Ma, Ruobing Bai, Sizhe Gao, Linlin Yang, Wenli Guo, Cong Zhang, Chengjun Kang, Yu Lan, Yanqiu Sun, Yonggao Zhang, Xigang Xiao, Yang Hou

**Affiliations:** 1 Department of Radiology, Shengjing Hospital of China Medical University, Shenyang, P.R. China; 2 Department of Radiology, Qinghai Provincial People’s Hospital, Qinghai, P.R. China; 3 Department of Radiology, The First Affiliated Hospital of Zhengzhou University, Zhengzhou, P.R. China; 4 Department of Radiology, The First Affiliated Hospital of Harbin Medical University, Harbin, P.R. China

**Keywords:** Dual-layer spectral-detector CT, MRI proton density fat fraction, Liver steatosis

## Abstract

**Objectives:**

To evaluate the diagnostic accuracy of liver dual-layer spectral-detector CT (SDCT) derived parameters of liver parenchyma for grading steatosis with reference to magnetic resonance imaging-based proton density fat fraction (MRI-PDFF).

**Methods:**

Altogether, 320 consecutive subjects who underwent MRI-PDFF and liver SDCT examinations were recruited and prospectively enrolled from four Chinese hospital centers. Participants were classified into normal (*n* = 152), mild steatosis (*n* = 110), and moderate/severe(mod/sev) steatosis (*n* = 58) groups based on MRI-PDFF. SDCT liver parameters were evaluated using conventional polychromatic CT images (CT_poly_), virtual mono-energetic images at 40 keV (CT_40kev_), the slope of the spectral attenuation curve (λ), the effective atomic number (Zeff), and liver to spleen attenuation ratio (L/S ratio). Linearity between SDCT liver parameters and MRI-PDFF was examined using Spearman correlation. Cutoff values for SDCT liver parameters in determining steatosis grades were identified using the area under the receiver-operating characteristic curve analyses.

**Results:**

SDCT liver parameters demonstrated a strong correlation with PDFF, particularly Zeff (r_s_ = −0.856; *p* < 0.001). Zeff achieved an area under the curve (AUC) of 0.930 for detecting the presence of steatosis with a sensitivity of 89.4%, a specificity of 82.4%, and an AUC of 0.983 for detecting mod/sev steatosis with a sensitivity of 93.1%, a specificity of 93.5%, the corresponding cutoff values were 7.12 and 6.94, respectively. Zeff also exhibited good diagnostic performance for liver steatosis grading in subgroups, independent of body mass index.

**Conclusion:**

SDCT liver parameters, particularly Zeff, exhibit excellent diagnostic accuracy for grading steatosis.

**Critical relevance statement:**

Dual-layer SDCT parameter, Zeff, as a more convenient and accurate imaging biomarker may serve as an alternative indicator for MRI-based proton density fat fraction, exploring the stage and prognosis of liver steatosis, and even metabolic risk assessment.

**Key Points:**

- Liver biopsy is the standard for grading liver steatosis, but is limited by its invasive nature.
- The diagnostic performance of liver steatosis using SDCT-Zeff outperforms conventional CT parameters.
- SDCT-Zeff accurately and noninvasively assessed the grade of liver steatosis.

**Graphical Abstract:**

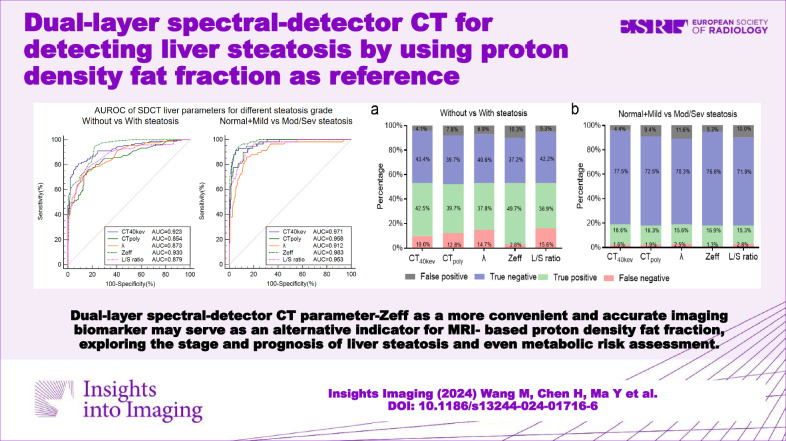

## Introduction

Metabolic-associated fatty liver disease (MAFLD) affects over 20% of the global population and keeps increasing over time [1, 2] The presence and severity of MAFLD have substantial implications for the development and incidence of chronic liver disease, and metabolic disorders [3, 4], and cardiovascular disease [1, 5, 6]. Steatosis is present throughout the disease’s progression and is strongly associated with obesity and other metabolic risk factors [3, 7]. Accurate assessment of steatosis is essential for managing patients with chronic liver disease in clinical practice and for conducting epidemiological and therapeutic studies in clinical research [8, 9]. Thus, a reliable, precise, and noninvasive imaging technology is essential for accurately determining liver steatosis extent and facilitating disease risk stratification.

Liver biopsy remains the gold standard for diagnosing and grading liver steatosis [10, 11]. However, this invasive method is not feasible for widespread use in assessing disease stage or determining progression or response to therapy, and it is subject to sampling variability [12, 13]. Although conventional CT is widely used in clinical settings as a screening tool, it has limited sensitivity for detecting mild fatty liver [14]. Previous studies have demonstrated that dual-energy computed tomography (DECT) attenuation exhibits excellent correlation with triglyceride content in phantom and animal studies [15], but it lacks validation in larger populations. Additionally, iodine uptake of liver parenchyma has shown good diagnostic performance for detecting liver steatosis [16], but it relies on contrast-enhanced CT images.

Non-enhanced CT has been used to assess liver steatosis in living liver donor candidates [17], as well as in cohort studies and clinical trials assessing the prevalence, natural history, prognosis, and treatment of liver steatosis [18, 19]. As an emerging and promising imaging technology, dual-layer spectral-detector computed tomography (SDCT) has been extensively employed, offering more imaging information than conventional CT through virtual single-energy X-ray imaging and material separation technology [20–24]. Recent research indicates that dual-energy subtraction imaging of spectral CT [25] and the decomposition algorithm of fat, iodine, and phantom material based on SDCT [26] are promising methods for quantitatively assessing hepatic fat quantification. However, prospective diagnostic studies adhering to the standards for reporting diagnostic accuracy studies guidelines [27] with SDCT have not been conducted. This study aimed to evaluate the diagnostic performance of SDCT liver parameters for detecting and classifying liver steatosis, using magnetic resonance imaging-based proton density fat fraction (MRI-PDFF) as the reference method [28]. Furthermore, we sought to identify clinically applicable cutoffs for SDCT liver parameters.

## Materials and methods

### Study participants

**Fig. 1:**
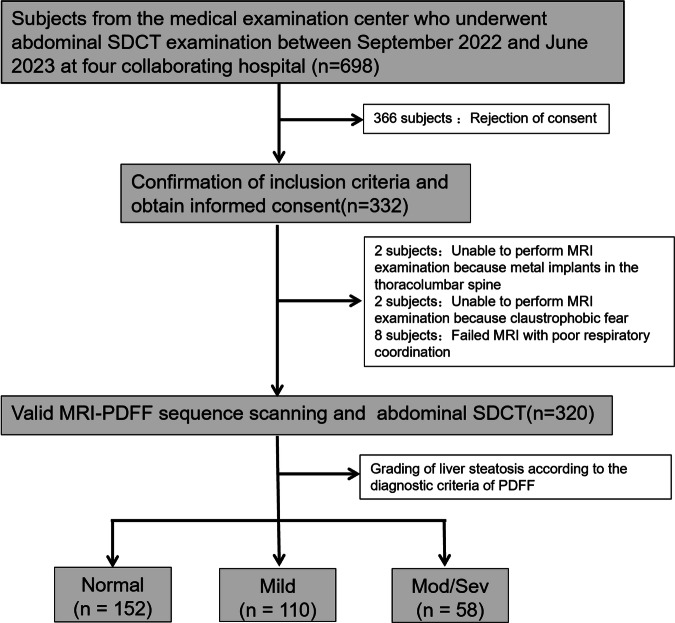
Flowchart of subject enrollment and the study design. Normal group, MRI-PDFF < 6.4%; mild group, 6.4% ≤ MRI-PDFF < 17.4%; mod/sev group, and MRI-PDFF ≥ 17.4%

The multicenter, prospective study received approval from the local hospital’s institutional review board (KYCS2022539). Prior to study registration, all subjects provided written informed consent. Inclusion criteria included subjects from the medical examination center aged 18 years or older. Exclusion criteria consisted of subjects with ascites and active implantable medical devices; individuals with a history of hepatic lobectomy, cirrhosis, or hemochromatosis; and those with poor breathing coordination and claustrophobia. Following informed agreement, a total of 332 consecutive participants were enrolled from September 2022 to June 2023 across four community hospital centers according to inclusion and exclusion criteria, and then we excluded two subjects with claustrophobic, two subjects with metal implants in the thoracolumbar spine, and eight subjects with poor respiratory coordination. Consequently, 320 subjects with valid MRI-PDFF sequence scanning and abdominal SDCT on the same day were further analyzed (Fig. 1). Among them, 242 subjects were from our main research hospital, and the other 78 subjects were from three other hospitals. Subjects’ laboratory data were measured within 1 month before and after CT scanning; if multiple measurements were taken, the laboratory data closest to the date of liver SDCT examination were taken.

### Reference standard: MRI proton density fat fraction technique

The subjects from four hospitals were all performed on the same 3-T magnetic resonance scanner (Philips Ingenia, Philips Healthcare, Best, the Netherlands) in each hospital, and we set the same scanning parameters. A triplane scout imaging gradient recalled echo sequence was first acquired, followed by a 3D multi-echo GRE sequence based on mDIXON technology. The mDIXON-QUANT sequence was acquired with the following settings: 6TES (first TE 0.97 ms, incremental TE 0.7 ms) and TR 5.6 ms, 3° flip angle, number of averaged signals 1, matrix size = 160 × 140, field of view = 400 × 350 mm, number of slices = 77, slice thickness = 3 mm. This sequence automatically generates water, fat, fat fraction, R2*, and T2* maps. Two experienced abdominal radiologists, with 6 and 8 years of experience respectively, measured PDFF value one week and six weeks after scanning, while blinded to clinical data. Liver circular regions of interest (ROI) located in the right lobe of the liver, approximately 300 mm^2^ (deviation < 10 mm^2^), were drawn while avoiding intrahepatic blood vessels, bile duct structures, focal liver lesions, and imaging artifacts. ROI placement was made side by side with CT (see later Fig. 5 for details) to match ROI placement as closely as possible.

Steatosis grade was defined as normal with MRI-PDFF < 6.4%, mild steatosis with 6.4% ≤ MRI-PDFF < 17.4%, and moderate and severe steatosis with MRI-PDFF ≥ 17.4%, according to the previous article [29].

### SDCT acquisition

All imaging was conducted using a 64-slice dual-layer SDCT scanner (IQon Spectral CT, Philips Healthcare, Best, the Netherlands). The scan range for all upper abdominal CTs spanned from the liver apex to 1 cm below the caudal end of the kidneys, utilizing an inspiratory breath-hold. Scanning parameters included a tube voltage of 120 kV, automatic tube current modulation technique (dose right index = 22 ref: 162 mAs), rotation time of 0.75 s, pitch of 1.172:1, and image thickness of 3.0 mm. Raw data were reconstructed employing a spectral iterative reconstruction algorithm (level-3), generating spectral base image datasets. The SDCT liver parameters were assessed by conventional polychromatic (120 kVp) images (CT_poly_), virtual monoenergetic images of 40 Kev (CT_40kev_) and 70 kev (CT_70kev_), the slope of the spectral attenuation curve [λ HU, λ HU = (CT_40kev_ − CT_70kev_)/30], effective atomic number of (Zeff), and liver to spleen attenuation ratio (L/S ratio). To manually delineate similar ROI on the multi-parameter images generated by SDCT, matching PDFF maps were established as closely as possible. Two radiologists—blinded for clinical data and PDFF results—measured liver parenchyma parameters. Simultaneously, spleen ROI was drawn on transverse sections through the right hepatic portal vein to estimate splenic attenuation value. The λ and L/S ratio was calculated from the mean after achieving consistency in liver and spleen measurements.

### Sample size estimation

Sample size estimation was executed using PASS 2021 version 21.0.3 software. No prior research has been conducted utilizing SDCT liver parameters; therefore, the sample size was determined based on the pre-experimental findings. Anticipating an area under the receiver operating characteristic curve (AUROC) of 0.850 for detecting any steatosis and 0.920 for moderate/severe (mod/sev) steatosis, with a (1-α) confidence interval (CI) and α set at 5%, and a 5% standard error level, a total of 282 subjects were required for SDCT and PDFF measurements. Assuming a 15% dropout rate, the final subjects count was established at 332.

### Statistical analysis

Statistical analyses were executed using SPSS Statistics V 26.0 (IBM Corporate, New York, USA) software. Non-normally distributed variables were presented as interquartile range and normally distributed variables were presented as mean ± standard deviation. The intraclass correlation coefficient (ICC) was employed to evaluate inter-observer agreement, while the Bland–Altman analysis assessed observer bias. The association between MRI-PDFF and SDCT liver parameters was ascertained using the Spearman correlation coefficient. The Kruskal–Wallis test was employed for comparing SDCT liver parameters across three different groups, whereas the Mann-Whitney U test was utilized for comparing across two groups. Receiver operating characteristic (ROC) curve analysis determined the diagnostic accuracy of SDCT liver parameters for identifying any steatosis and mod/sev steatosis. Area under the curve (AUC) comparisons followed the methodology proposed by DeLong et al *p* values < 0.05 indicated a statistically significant difference.

## Results

### Study population information

A total of 320 consecutive subjects participated in the study. Table 1 displays the characteristics and laboratory data of the enrolled subjects. No adverse events were reported in association with the SDCT and MRI examination.

**Table 1:** Demographic characteristics of different liver steatosis groups

Parameter	Total, (*n* = 320)	Normal, (*n* = 152)	Mild, (*n* = 110)	Mod/sev, (*n* = 58)	P1	P2	P3
Gender (F:M)	200:120	107:45	58:52	35:23	0.013	0.006	0.708
Age (y)	45.0 (37.0–54.0) *	44.0 (36.0–53.0) *	46.0 (38.0–53.0) *	48.0 (35.0–59.0) *	0.367	0.209	0.252
BMI (kg/m^2^)	26.0 (24.2–27.8) *	24.6 (22.5–27.0) *	26.3 (25.3–28.3) *	27.3 (26.0–31.1) *	< 0.001	< 0.001	< 0.001
ALT (IU/L)	21.0 (14.0–31.0) *	17.0 (13.0–26.0) *	24.0 (17.0–34.0) *	28.5 (18.0–37.0) *	< 0.001	< 0.001	< 0.001
AST (IU/L)	19.0 (16.0–25.0) *	18.0 (15.0–22.0) *	20.0 (17.0–27.0) *	22.5 (18.0–28.0) *	< 0.001	< 0.001	< 0.001
GGT (IU/L)	24.0 (16.0–37.0) *	21.0 (15.0–30.0) *	27.0 (19.0–43.0) *	33.0 (22.0–44.0) *	< 0.001	< 0.001	< 0.001
Triglyceride (mmol/L)	1.3 (1.0–2.0) *	1.2 (0.9–1.7) *	1.2 (1.0–2.3) *	2.0 (1.5–2.8) *	< 0.001	< 0.001	< 0.001
HDL (mmol/L)	1.3 (1.1–1.6) *	1.4 (1.1–1.6) *	1.3 (1.1–1.6) *	1.0 (0.8–1.2) *	< 0.001	0.001	< 0.001
LDL (mmol/L)	3.0 (2.3–3.6) *	2.8 (2.2–3.6) *	3.1 (2.3–3.6) *	3.4 (2.4–4.0) *	0.032	0.092	0.011
FBG (mmol/L)	5.8 (4.7–7.0) *	4.7 (4.3–5.6) *	6.4 (5.3–7.6) *	6.9 (6.4–8.5) *	< 0.001	< 0.001	< 0.001
HbA1C (%)	6.1 (5.6–6.8) *	5.8 (5.4–6.4) *	6.2 (5.7–7.1) *	6.4 (6.1–7.9) *	< 0.001	< 0.001	< 0.001
Hypertension, *n* (%)	30.9	25.7	33.6	39.7	0.110	0.052	0.112
Diabetes mellitus, *n* (%)	34.7	23.7	39.1	55.2	< 0.001	< 0.001	< 0.001
Alcohol drinker, *n* (%)	38.8	34.9	41.8	43.1	0.394	0.175	0.452

*BMI* body mass index, *ALT* alanine transaminase, *AST* aspartate transaminase, *GGT* gamma-glutamyl transpeptidase, *HDL* high-density lipoprotein, *P1* *p* value of normal vs mild vs mod/sev steatosis, *P2* *p* value of without vs with steatosis, *P3* *p* value of mild vs mod/sev steatosis

* Median with 25th and 75th percentiles

### Measurement agreement assessment

Excellent inter-observer agreement was observed for PDFF measurements (ICC = 0.976; 95% CI: 0.970–0.980), CT_40kev_ measurements (ICC = 0.974; 95% CI: 0.967–0.979), CT_poly_ measurements (ICC = 0.917; 95% CI: 0.898–0.933), Zeff measurements (ICC = 0.960; 95% CI: 0.951–0.968). We also analyzed the systematic bias of different CT parameters between two different viewers (Supplementary Fig. 1). No statistically significant bias was found among the quantitative parameters, and the number of large deviations was controlled within 5%. Consequently, the mean values of the two measurements were used for all calculations and statistical comparisons.

### SDCT liver parameters distribution and correlation assessment

**Fig. 2:**
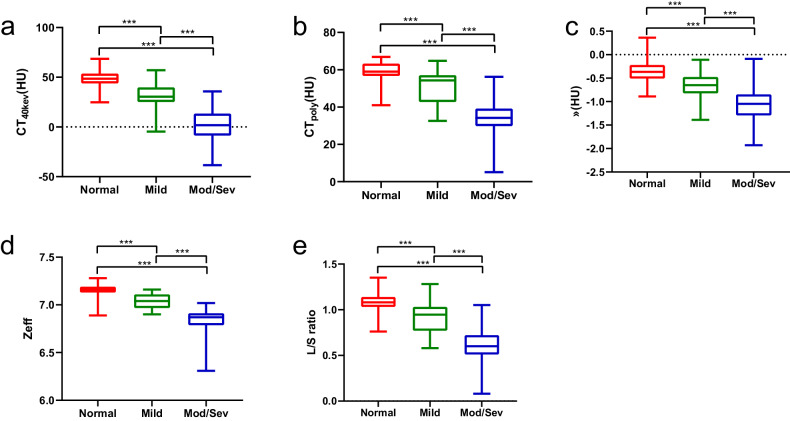
SDCT liver parameters distribution among different steatosis grades. (**a**) CT_40kev_, (**b**) CT_poly_, (**c**) λ, (**d**) Zeff, and (**e**) L/S ratio (***means *p* < 0.001)

**Fig. 3:**
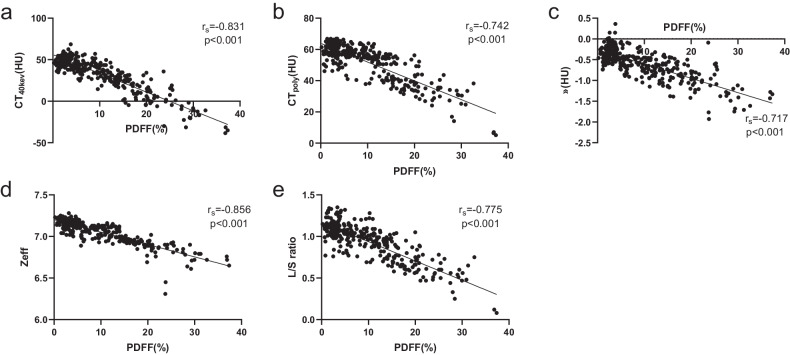
Linearity of SDCT liver parameters against MRI-PDFF. (**a**) CT_40kev_, (**b**) CT_poly_, (**c**) λ, (**d**) Zeff, and (**e**) L/S ratio

The distribution of SDCT liver parameters among different steatosis grading groups was analyzed, as shown in Table 2 and Fig. 2. The SDCT liver parameters consistently decreased with increasing liver steatosis grade, and the differences among normal, mild, and mod/sev groups were statistically significant (all *p* < 0.001). Linearity between MRI-PDFF and SDCT liver parameters is demonstrated in Fig. 3. Scatter plots revealed significant correlations between liver multi-parameters and PDFF in the general population, particularly Zeff (r_s_ = −0.856; *p* < 0 .001).

**Table 2:** PDFF and SDCT characteristics of different liver steatosis groups

Parameter	Total	Normal, (*n* = 152)	Mild, (*n* = 110)	Mod/sev, (*n* = 58)	P1	P2	P3
PDFF (%)	7.4 (3.2–15.1) *	3.1 (2.2–4.0) *	12.0 (9.6–14.4) *	22.0 (20.0–27.5) *	< 0.001	< 0.001	< 0.001
CT_40kev_ (HU)	38.8 (24.7–48.8) *	48.5 (43.9–53.6) *	30.5 (25.1–39.7) *	1.8 (−8.5–13.3) *	< 0.001	< 0.001	< 0.001
λ (HU)	−0.60 ± 0.37	−0.37 ± 0.22	−0.68 ± 0.25	−1.08 ± 0.35	< 0.001	< 0.001	< 0.001
Zeff	7.09 (6.96–7.16) *	7.16 (7.13–7.19) *	7.04 (6.97–7.11) *	6.87 (6.79–6.91) *	< 0.001	< 0.001	< 0.001
CT_poly_ (HU)	56.0 (43.8–60.5) *	59.0 (56.7–63.3) *	54.3 (42.7–57.1) *	34.2 (29.9–39.2) *	< 0.001	< 0.001	< 0.001
L/S ratio	1.00 (0.78–1.09) *	1.08 (1.03–1.14) *	0.95 (0.77–1.03) *	0.60 (0.51–0.72) *	< 0.001	< 0.001	< 0.001

*PDFF* proton density fat fraction, *P1* *p* value of normal vs mild vs mod/sev steatosis, *P2* *p* value of without vs with steatosis, *P3* *p* value of mild vs mod/sev steatosis

* Median with 25th and 75th percentiles

### Diagnostic performance of SDCT liver parameters by using MRI-PDFF as the reference technique

**Fig. 4:**
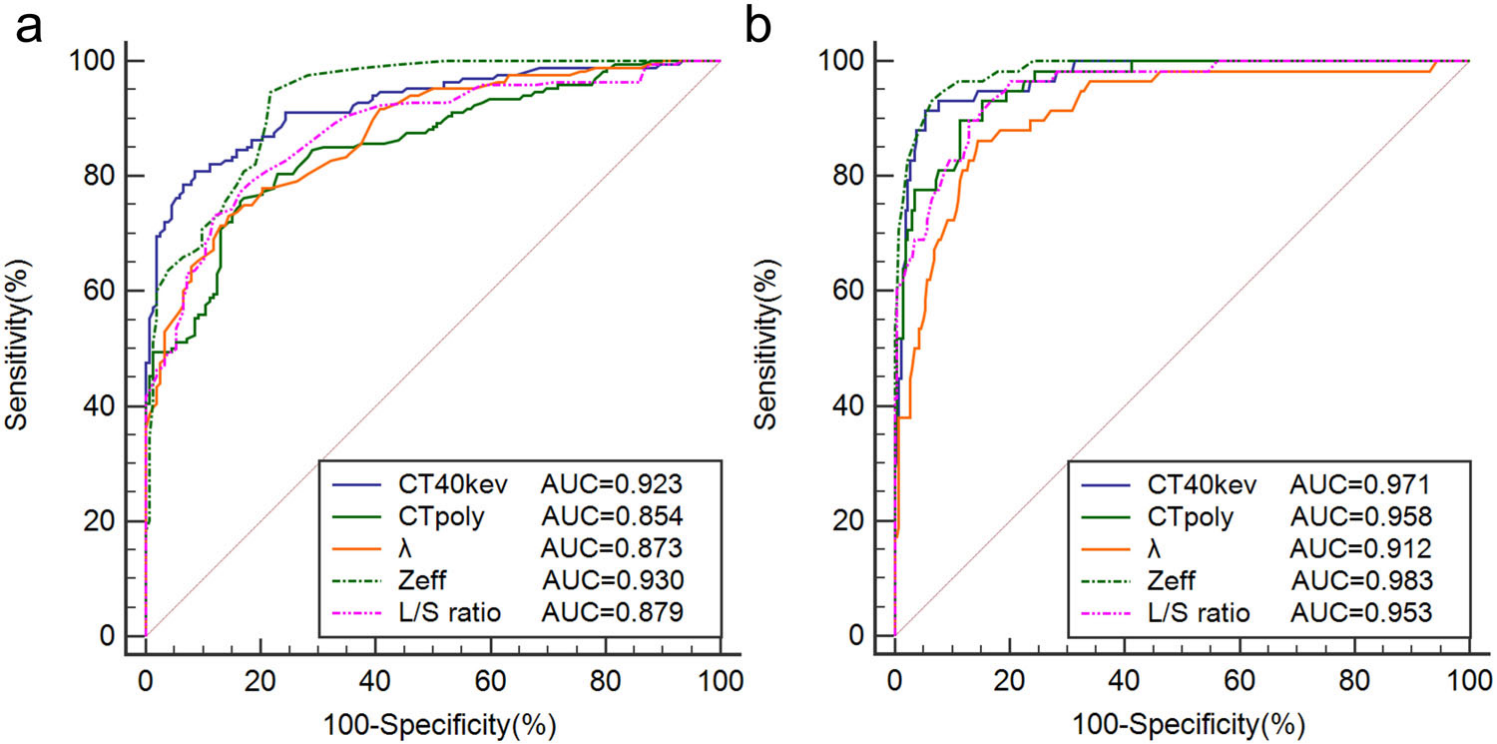
Results of ROC curve analysis for SDCT liver parameters as criteria for diagnosis of different grades of liver steatosis. **a** AUROCs of CT_40kev_, CT_poly_, λ, Zeff, and L/S ratio for diagnosing the presence of steatosis. **b** AUROCs of CT_40kev_, CT_poly_, λ, Zeff, and L/S ratio for diagnosing mod/sev steatosis

Table 3 and Fig. 4 analyze the AUROCs and diagnostic performance of SDCT liver parameters, and the cutoff values using Youden’s Index for the presence of steatosis and mod/sev steatosis. Zeff (≤ 7.12) achieved better diagnostic performance for diagnosing the presence of steatosis from normal than did CT_poly_ (≤ 55.6 HU) (AUC: 0.930 vs 0.854, *p* < 0.001) and L/S ratio (≤ 0.97) (AUC: 0.930 vs 0.879, *p* = 0.008). Zeff (≤ 6.94) also achieved better diagnostic performance for diagnosing mod/sev steatosis from normal and mild steatosis than did CT_poly_ (≤ 44.0 HU) (AUC: 0.983 vs 0.958, *p* = 0.035) and L/S ratio (≤ 0.80) (AUC: 0.983 vs 0.955, *p* = 0.028).

**Table 3:** Diagnostic ability and optimal cutoff values of multi-parameter liver spectral CT metrics for grading liver steatosis using MRI-PDFF as reference

Steatosis grade	Parameters	AUC	Cutoff	Sensitivity (%)	Specificity (%)	PPV (%)	NPV (%)
Without vs with steatosis	CT_40kev_ (HU)	0.923 (0.889–0.950)	≤ 37.4	81.0 (74.2–86.6)	91.5 (85.8–95.4)	91.3 (86.1–94.6)	81.3 (76.0–85.6)
	CT_poly_ (HU)	0.854 (0.810–0.890)	≤ 55.6	75.6 (68.4–81.9)	83.6 (76.7–89.1)	83.6 (77.8–88.0)	75.6 (70.2–80.3)
	λ (HU)	0.873 (0.831–0.907)	≤ −0.59	73.2 (65.8–79.7)	85.5 (78.9–90.7)	84.8 (79.0–89.3)	74.3 (69.0–78.9)
	Zeff	0.930 (0.896–0.955)	≤ 7.12	89.4 (84.1–93.4)	82.4 (74.8–88.5)	88.0 (83.5–91.4)	84.4 (78.0–89.2)
	L/S ratio	0.879 (0.838–0.912)	≤ 0.97	73.2 (65.8–79.7)	88.2 (81.9–92.8)	87.2 (81.4–91.4)	74.9 (69.7–79.4)
Normal + mild vs mod/sev steatosis	CT_40kev_ (HU)	0.971 (0.946–0.986)	≤ 19.7	91.4 (81.0–97.1)	94.7 (91.2–97.0)	79.1 (69.3–86.4)	98.0 (95.5–99.1)
	CT_poly_ (HU)	0.958 (0.930–0.977)	≤ 44.0	89.7 (78.8–96.1)	88.6 (84.1–92.1)	63.4 (55.0–71.1)	97.5 (94.8–98.8)
	λ (HU)	0.912 (0.876–0.841)	≤ −0.78	68.1 (57.5–77.5)	74.5 (64.7–82.8)	71.3 (63.2–78.2)	71.6 (64.6–77.6)
	Zeff	0.983 (0.962–0.994)	≤ 6.94	93.1 (83.3–98.1)	93.5 (89.8–96.2)	76.1 (66.6–83.5)	98.4 (96.0–99.4)
	L/S ratio	0.955 (0.926–0.975)	≤ 0.80	89.7 (78.8–96.1)	87.0 (82.3–90.8)	60.5 (52.5–67.9)	97.4 (94.7–98.8)

*PPV* positive predictive value, *NPV* negative predictive value

**Fig. 5:**
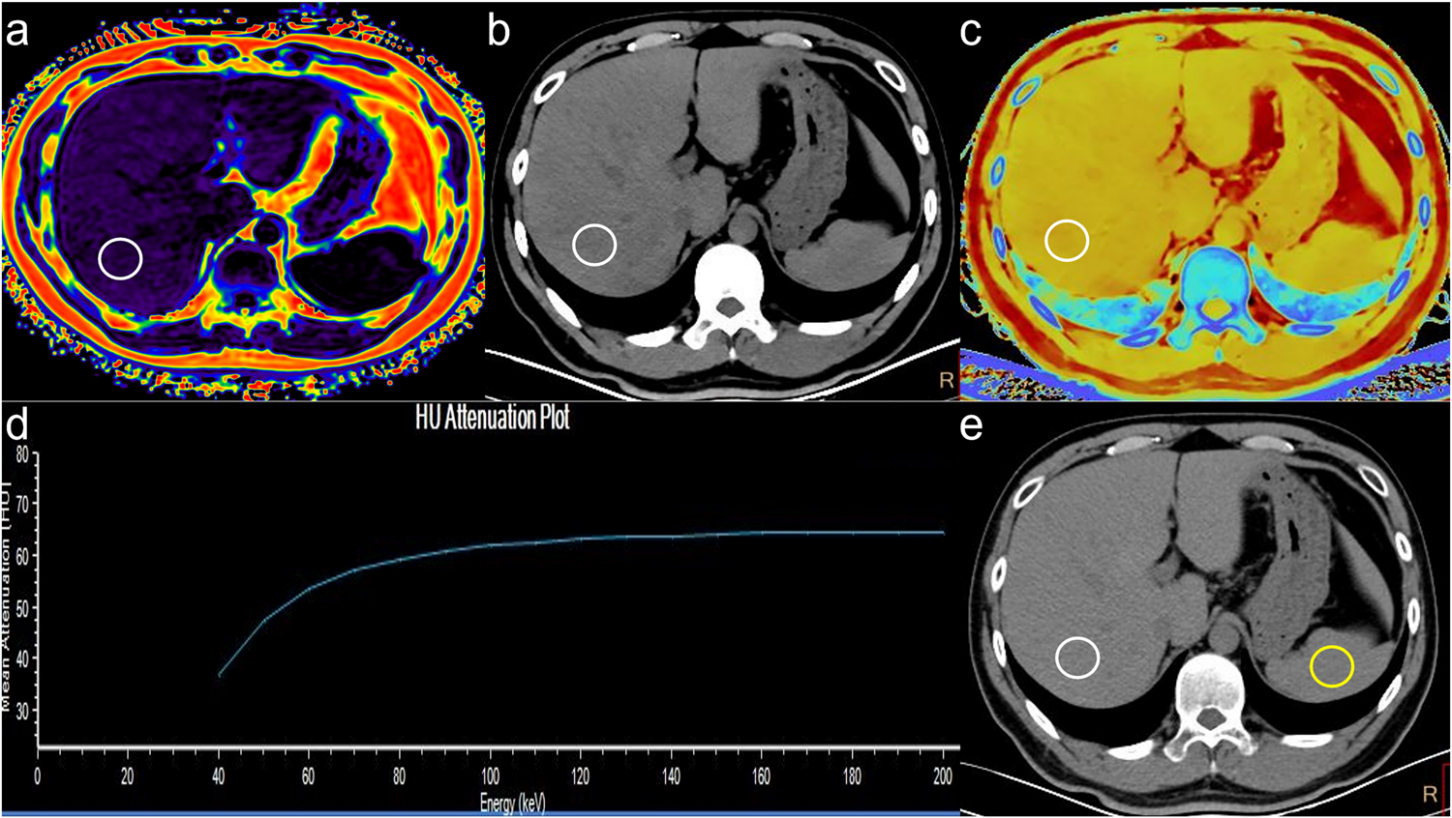
Liver PDFF images and SDCT images (transverse CT sections; 3-mm thick) from a 41-year-old male (BMI 28.55 kg/m^2^) with mild steatosis. The size of the ROI was set to 300 mm^2^. Liver circular ROI is located at the level of the portal right branch emanating from the main portal vein, outlined with a white solid line. The spleen circular ROI is delineated at the same axial level, outlined with a yellow solid line, for the purpose of calculating the L/S ratio. (**a**) Liver PDFF image, PDFF = 7.3%, (**b**) CT_40kev_ image, CT_40kev_ = 38.10 HU, (**c**) Zeff image, Zeff = 7.06, (**d**) λ image, λ = −0.63, and (**e**) CT_poly_ image, CT_poly_ = 58.00 HU, L/S ratio = 1.13, Zeff image consistent with PDFF grade, while CT_poly_ and L/S ratio categorizes it as normal group

**Fig. 6:**
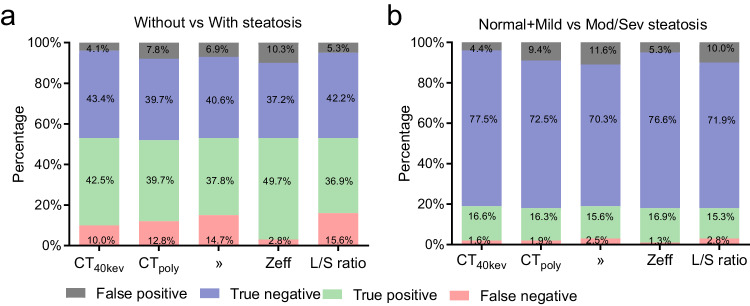
Results of diagnostic performance of SDCT liver parameters for diagnosing different grades of liver steatosis. **a** Without vs With steatosis. **b** Normal + Mild vs Mod/Sev steatosis. Color coding represents the proportion of false negative, true positive, true negative, and false positive. Zeff increases diagnostic performance by minimizing the proportion of false positive and maximizing the proportion of true negative

As shown in Fig. 5 a representative case for mild steatosis was correctly diagnosed by SDCT-Zeff, while being wrongly diagnosed as a normal person by conventional CT liver parameters(CT_poly_ and L/S ratio). The diagnostic performance of different SDCT liver parameters in distinguishing liver steatosis grades is shown in Fig. 6. USing CT_poly_ as a diagnostic parameter, 168 subjects (52.5%) were diagnosed as normal, 70 subjects (21.9%) as mild, and 82 subjects (25.6%) as mod/sev steatosis, resulting in 16 subjects (5.0%) with mild liver steatosis being misdiagnosed as normal and 24 subjects (7.5%) with mild liver steatosis being misdiagnosed as mod/sev steatosis. However, using SDCT-Zeff as a diagnostic parameter, 128 subjects (40.0%) were diagnosed as normal, 121 subjects (37.8%) as mild, and 71 subjects (22.2%) as mod/sev steatosis. By contrast, only 24 subjects (7.5%) were misdiagnosed as the presence of steatosis, indicating that Zeff increased the accuracy of diagnosis, with an accuracy of 86.9% in differentiating any steatosis and 93.5% in differentiating mod/sev steatosis.

### Assessment of diagnostic ability of Zeff for grading steatosis in subgroups

To evaluate the diagnostic performance of Zeff, we further analyzed its diagnostic accuracy in different subgroups, as presented in Table 4. The results demonstrated that for diagnosing any steatosis and mod/sev steatosis in high BMI (> 25.0 kg/cm^2^), and low BMI groups, the AUROC of Zeff consistently exceeded 0.90, with sensitivity greater than 90.00% and specificity exceeded 75.00%.

**Table 4:** Diagnostic performance of SDCT parameters for grading hepatic steatosis in the subgroup analysis

Steatosis grade	Number	Cutoff	AUC, (95% CI)	Sensitivity, (%)	Specificity, (%)	PPV, (%)	NPV, (%)
BMI-low (≤ 24.9) (kg/m^2^)							
Normal vs any steatosis	83:112	≤ 7.12	0.919 (0.852–0.962)	93.1 (77.2–99.2)	80.7 (70.6–88.6)	62.8 (51.8–72.6)	97.1 (89.8–99.2)
Normal + mild vs mod/sev steatosis	106:6	≤ 6.94	0.998 (0.963–1.000)	100.0 (54.1–100.0)	98.1 (93.4–99.8)	75.0 (43.2–92.2)	100.0
BMI-high (> 25.0) (kg/m^2^)							
Normal vs any steatosis	69:208	≤ 7.12	0.922 (0.877–0.955)	95.0 (89.9–98.0)	75.4 (63.5–84.9)	88.6 (83.7–92.2)	88.1 (78.1–93.9)
Normal + mild vs mod/sev steatosis	156:52	≤ 6.94	0.974 (0.942–0.991)	92.3 (81.5–97.9)	90.4 (84.6–94.5)	76.2 (66.3–83.9)	97.2 (93.2–98.9)

*PPV* positive predictive value, *NPV* negative predictive value

## Discussion

Our study showed that SDCT liver parameters perform better in differentiating the presence of liver steatosis and mod/sev steatosis than conventional CT parameters, especially Zeff with the best sensitivity, and the corresponding thresholds are 7.12 and 6.94, respectively. SDCT-Zeff also shows good repeatability and is not affected by BMI, which is expected to provide noninvasive guidance for the early detection of high-risk metabolic diseases associated with liver steatosis.

MRI-PDFF has become the reference standard for noninvasive quantification of liver steatosis. However, its implementation for widespread fatty liver screening and longitudinal studies is impractical in most hospitals. SDCT is an increasingly widely used dual-energy CT technology that utilizes two layers of detectors to simultaneously achieve low-energy and high-energy data from all subjects using standard CT protocols. It enables comprehensive tissue characterization by providing spectral and quantitative virtual mono-energy image results at a wide range of energy levels, including λ HU and Zeff [30, 31]. To our knowledge, this is the first study to evaluate liver steatosis in a population using quantitative parameters obtained with non-enhanced SDCT. The results of this study show that Zeff had a better diagnostic performance than other SDCT liver parameters for the diagnosis of the presence of any steatosis and mod/sev steatosis. The potential mechanism may be that the Zeff value reflects the characteristics of the composite atoms for the mixture of various cells, compounds, or materials, which may facilitate the differentiation of different tissue types with very similar electron densities and the same CT attenuation values [32, 33]. Several studies have shown that Zeff can describe tissue characterization [34, 35], while CT value represents the combination of atomic number of the materials and their density, so it is challenging for CT number to differentiate and classify different types of tissues [36].

Previous studies [15] have shown that CT attenuation is superior at quantifying triglyceride content than DECT fat density and Zeff measurements, therefore, does not enable quantification of liver fat in vivo beyond what conventional CT can provide. This may be in contradiction with our research results. However, the correlation between Zeff and MRI-FF (*r*^2^ = 0.67, *p* < 0.001) is lower than our research results (|r_s_| = 0.856, *p* < 0.001). Moreover, the research is based on the results of animal experiments and has not been verified in people. Tomoko Hyodo [37] showed that quantitative liver fat based on a multi-material decomposition (MMD) algorithm is a reproducible and accurate imaging method. However, the development of the MMD algorithm relies on GE dual-energy CT equipment, while the substance decomposition algorithm on SDCT has not yet been widely applied in clinical practice at present [26]. Beck’s [16] research concludes that the measurement of iodine concentration on DECT can provide an appropriate method for the detection of liver steatosis in quantitative iodine images and the performance is comparable to our study based on SDCT scan (AUC: 0.937 vs 0.930), while our study did not require additional radiation exposure and contrast agent load. Additionally, the existence and severity of steatosis may be sensitively and precisely diagnosed, establishing the foundation for widespread clinical application.

Previous studies have shown that BMI may affect the assessment of liver steatosis, especially the controlled attenuation parameter (CAP) in detecting mod/sev steatosis, therefore, the M probe was often used in participants with BMI < 30 kg/m^2,^ and XL probe in participants with BMI ≥ 30 kg/m^2^, however, we discovered that the M probe under-quantifies CAP values in comparison to the XL probe in the same patients. Thus, when interpreting the CAP value of the patients with liver steatosis, the type of probes should be considered [38]. However, subgroup study results revealed that Zeff retained good differential diagnostic capacity across BMI groups. This may be attributed to the difference in the imaging principles of CT and ultrasound (US) scans [39], which means that the US is influenced by factors such as probe type and depth from skin to liver capsule. Based on the above study results, we concluded that the liver multi-parameter metrics, extensively utilized as a quantitative method for assessing steatosis in clinical practice, exhibited excellent technical performance. When compared to the US and MRI, the feasibility is up to 100%. The utilization of abdominal CT scans significantly surpasses that of MRI, especially among the population undergoing physical examinations. Furthermore, the use of dual-energy CT is becoming increasingly prevalent. Thus, SDCT can serve as an effective opportunistic screening tool for the initial identification and quantification of liver steatosis.

There are several limitations to our study. Firstly, none of the liver steatosis has been assessed by liver biopsy, because it has become unethical to undertake liver biopsy only for the purpose of assessing hepatic steatosis. Instead, we used the MRI-PDFF as a reference, which was a recommended and validated method to quantify steatosis in clinical practice. Secondly, the proportion of subjects with different grades of steatosis is uneven in our study, but the enrolled population roughly meets the proportion of different grades of steatosis in the Chinese population [40]. Thirdly, although we set the same parameters for scanning equipment of the same model in different hospitals, not all patients completed the examination on the same equipment. Fourthly, despite our best efforts to match the ROI selection in our investigation, there may still be some measurement deviation due to the different scanning slice thicknesses between CT and MRI-PDFF.

## Conclusion

With Reference to MRI-PDFF, Zeff based on SDCT scan has superior detection and grading abilities for liver steatosis compared to CT_poly_ values and L/S ratio of conventional CT, and the thresholds for detecting the presence of steatosis and mod/sev steatosis are 7.12 and 6.94, respectively. This may bring new opportunities for noninvasive fatty liver detection and risk classification of metabolic disorders associated with fatty liver in clinical practice and research.

### Supplementary information

Supplementary fig.1

Supplementary fig.1 legend

## Data Availability

The original data generated in this study can be requested from the corresponding author. For any researchers interested in obtaining these data, we encourage you to contact the corresponding author via email at houy2@sj-hospital.org to discuss the specific conditions for data access and possible measures for privacy protection.

## Abbreviations

AUC: Area under the curve
AUROC: Area under the receiver operating characteristic curve
BMI: Body mass index
CAP: Controlled attenuation parameter
CI: Confidence interval
DECT: Dual-energy computed tomography
ICC: Intraclass correlation coefficient
MAFLD: Metabolic-associated fatty liver disease
MRI-PDFF: Magnetic resonance imaging-based proton density fat fraction
ROC: Receiver operating characteristic
ROI: Regions of interest
SDCT: Spectral-detector computed tomography
US: Ultrasound
