# Supplementary figures and images for: Dual-layer spectral-detector CT for detecting liver steatosis by using proton density fat fraction as reference
Source: Insights Imaging. 2024 Aug 15;15:210. doi: 10.1186/s13244-024-01716-6 (PMC11327236; doi:10.1186/s13244-024-01716-6)

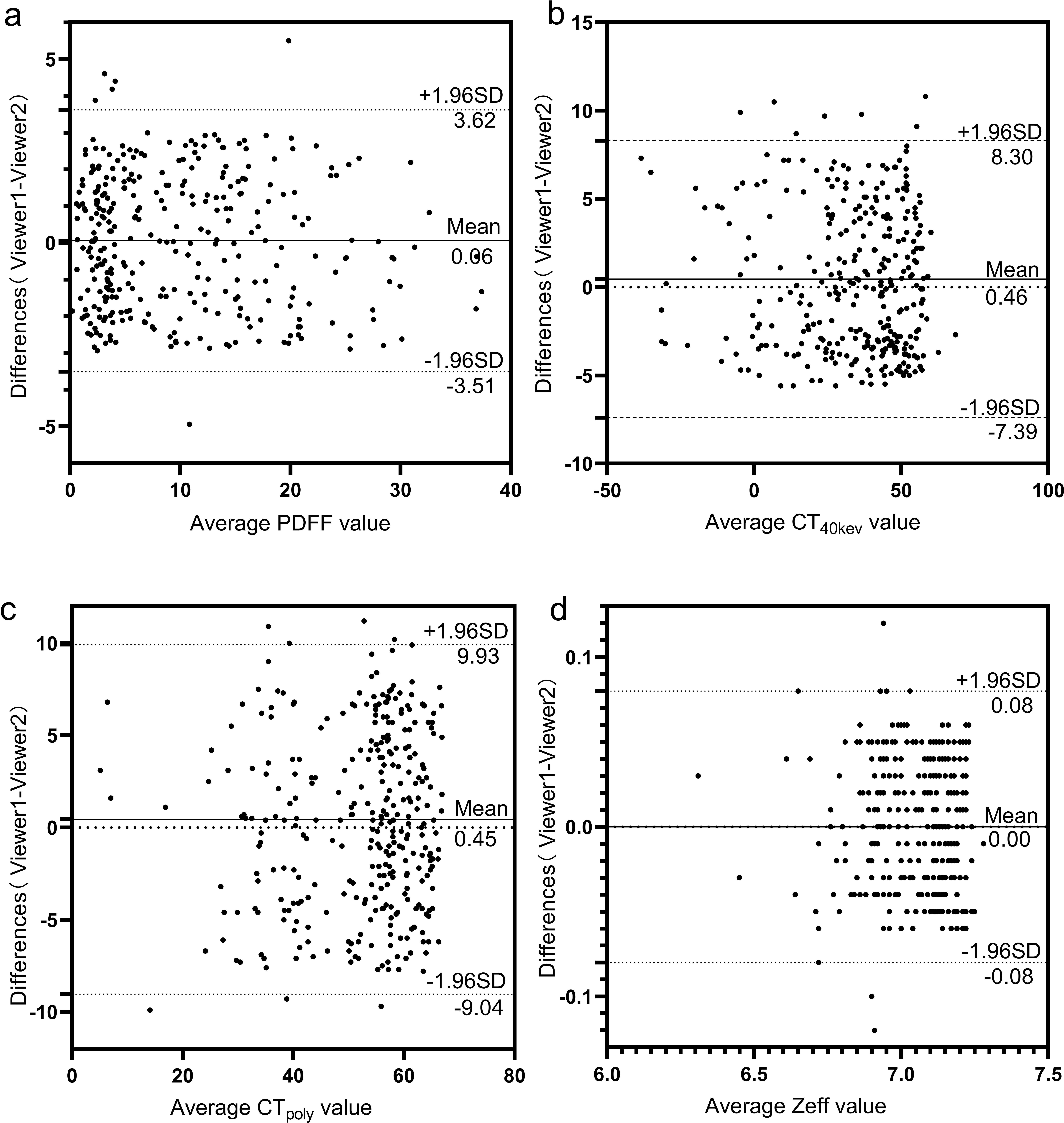

Supplement: Supplementary file 1 — Supplementary fig.1 [file 13244_2024_1716_MOESM1_ESM.jpg]
