# Supplementary material for: Dual-layer spectral-detector CT for detecting liver steatosis by using proton density fat fraction as reference
Source: Insights Imaging. 2024 Aug 15;15:210. doi: 10.1186/s13244-024-01716-6 (PMC11327236; doi:10.1186/s13244-024-01716-6)
Supplement: Supplementary file 2 — Supplementary fig.1 legend [file 13244_2024_1716_MOESM2_ESM.docx]

Supplementary figure 1. Bland-Altman plot of SDCT liver parameters for two viewers. (a) PDFF. (b) CT_40kev_. (c) CT_poly_. (d) Zeff.
